# Bis(ethanamidinium) (1,10-phen­an­thro­line-2,9-dicarboxyl­ato)manganate(II) hepta­hydrate

**DOI:** 10.1107/S1600536812048350

**Published:** 2012-12-05

**Authors:** Yan-Li Miao, Hong-Bo Wang, Wen-Dong Song

**Affiliations:** a College of Science, Guang Dong Ocean University, Zhanjiang 524088, People’s Republic of China

## Abstract

In the title complex, (C_2_H_7_N_2_)_2_[Mn(C_14_H_6_N_2_O_4_)_2_]·7H_2_O, the Mn^II^ atom is coordinated by four N atoms and four O atoms from two 1,10-phenanthroline-2,9-dicarboxyl­ate ligands in a distorted dodeca­hedral geometry. The double negative charge is balanced by two ethanamidinium cations. A three-dimensional supra­molecular structure is formed through N—H⋯O and O—H⋯O hydrogen bonds and π–π stacking inter­actions [centroid–centroid distance = 3.553 (2) Å].

## Related literature

For general background to 1,10-phenanthroline derivatives, see: Kaes *et al.* (2000 ▶); Albores & Rentschler (2008 ▶); Sreerama & Pal (2004 ▶) and to 1,10-phenanthroline-2,9-dicarboxyl­ate (H_2_phenda), see: Dean *et al.* (2008 ▶); Gephart *et al.* (2008 ▶); Moghimi *et al.* (2005 ▶); Fan *et al.* (2008 ▶). For the synthesis, see: Chandler *et al.* (1981 ▶). 
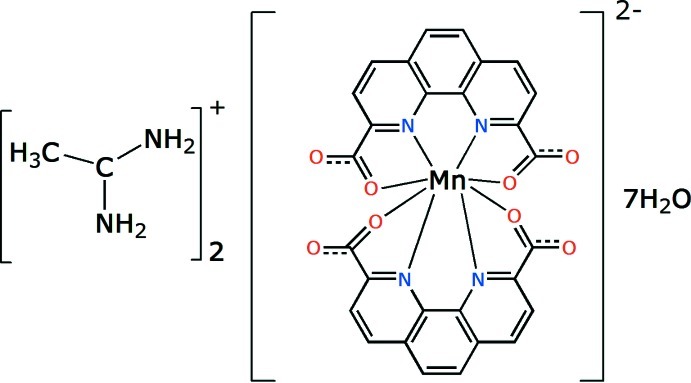


## Experimental

### 

#### Crystal data

(C_2_H_7_N_2_)_2_[Mn(C_14_H_6_N_2_O_4_)_2_]·7H_2_O
*M* *_r_* = 831.66
Triclinic, 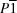

*a* = 9.6330 (6) Å
*b* = 13.8174 (7) Å
*c* = 15.4828 (8) Å
α = 66.151 (5)°
β = 78.949 (5)°
γ = 75.397 (5)°
*V* = 1814.56 (19) Å^3^
*Z* = 2
Cu *K*α radiation
μ = 3.69 mm^−1^
*T* = 150 K
0.29 × 0.26 × 0.16 mm

#### Data collection

Agilent Gemini S Ultra CCD diffractometer
Absorption correction: multi-scan (Blessing, 1995 ▶) *T*_min_ = 0.395, *T*_max_ = 0.554
10581 measured reflections
5310 independent reflections
4336 reflections with *I* > 2σ(*I*)
*R*_int_ = 0.024
θ_max_ = 60.0°

#### Refinement

*R*[*F* ^2^ > 2σ(*F* ^2^)] = 0.037
*wR*(*F* ^2^) = 0.102
*S* = 0.95
5310 reflections
507 parameters
H-atom parameters constrained
Δρ_max_ = 0.49 e Å^−3^
Δρ_min_ = −0.38 e Å^−3^

### 

Data collection: *CrysAlis CCD* (Oxford Diffraction, 2006 ▶); cell refinement: *CrysAlis RED* (Oxford Diffraction, 2006 ▶); data reduction: *CrysAlis RED*; program(s) used to solve structure: *SHELXS97* (Sheldrick, 2008 ▶); program(s) used to refine structure: *SHELXL97* (Sheldrick, 2008 ▶); molecular graphics: *SHELXTL* (Sheldrick, 2008 ▶); software used to prepare material for publication: *SHELXTL*.

## Supplementary Material

Crystal structure: contains datablock(s) I, global. DOI: 10.1107/S1600536812048350/fi2126sup1.cif

Supplementary material file. DOI: 10.1107/S1600536812048350/fi2126Isup2.cdx

Structure factors: contains datablock(s) I. DOI: 10.1107/S1600536812048350/fi2126Isup3.hkl

Supplementary material file. DOI: 10.1107/S1600536812048350/fi2126Isup4.cdx

Additional supplementary materials:  crystallographic information; 3D view; checkCIF report

## Figures and Tables

**Table 1:** Hydrogen-bond geometry (Å, °)

*D*—H⋯*A*	*D*—H	H⋯*A*	*D*⋯*A*	*D*—H⋯*A*
N5—H5*A*⋯O1*W*	0.90	1.93	2.792 (4)	159
N5—H5*B*⋯O2	0.90	1.93	2.797 (3)	161
N6—H6*A*⋯O1*W*	0.90	2.19	2.938 (4)	140
N6—H6*B*⋯O7*W* ^i^	0.90	1.94	2.830 (3)	169
N7—H7*A*⋯O8	0.90	1.96	2.840 (3)	167
N7—H7*B*⋯O1^ii^	0.90	1.93	2.812 (3)	168
N8—H8*A*⋯O3*W*	0.90	2.05	2.941 (3)	172
N8—H8*B*⋯O2*W* ^iii^	0.90	1.98	2.871 (3)	173
O1*W*—H1*WB*⋯O6^iv^	0.85	1.93	2.780 (3)	178
O1*W*—H1*WA*⋯O2*W*	0.85	1.98	2.826 (3)	171
O2*W*—H2*WA*⋯O4^v^	0.85	1.92	2.709 (3)	154
O2*W*—H2*WB*⋯O3^ii^	0.85	1.90	2.753 (3)	177
O3*W*—H3*WA*⋯O8	0.85	1.87	2.687 (3)	161
O3*W*—H3*WB*⋯O2^iii^	0.85	1.94	2.741 (3)	156
O4*W*—H4*WA*⋯O3*W*	0.85	2.18	2.996 (3)	161
O4*W*—H4*WB*⋯O7	0.85	1.98	2.808 (3)	164
O5*W*—H5*WA*⋯O4*W*	0.85	1.99	2.828 (3)	168
O5*W*—H5*WB*⋯O6*W*	0.85	1.99	2.783 (3)	154
O6*W*—H6*WA*⋯O6^vi^	0.85	1.89	2.737 (3)	174
O6*W*—H6*WB*⋯O5*W* ^vii^	0.85	2.03	2.826 (4)	155
O7*W*—H7*WA*⋯O6*W*	0.85	1.96	2.760 (3)	157
O7*W*—H7*WB*⋯O5^vi^	0.85	2.02	2.865 (3)	173

Symmetry codes: (i) 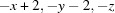; (ii) 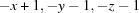; (iii) 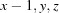; (iv) 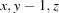; (v) 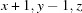; (vi) 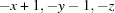; (vii) 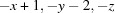.

**Fig. 1.**
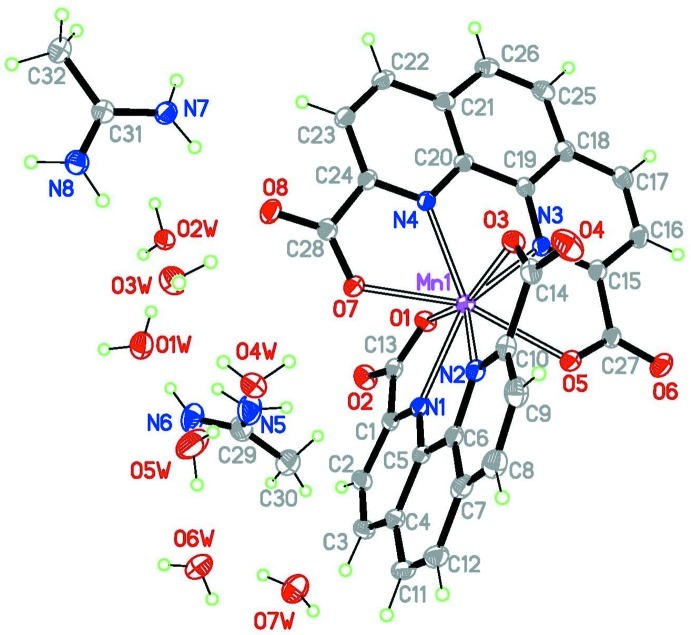
The molecular structure of (I), showing 30% probability displacement ellipsoids.

**Fig. 2.**
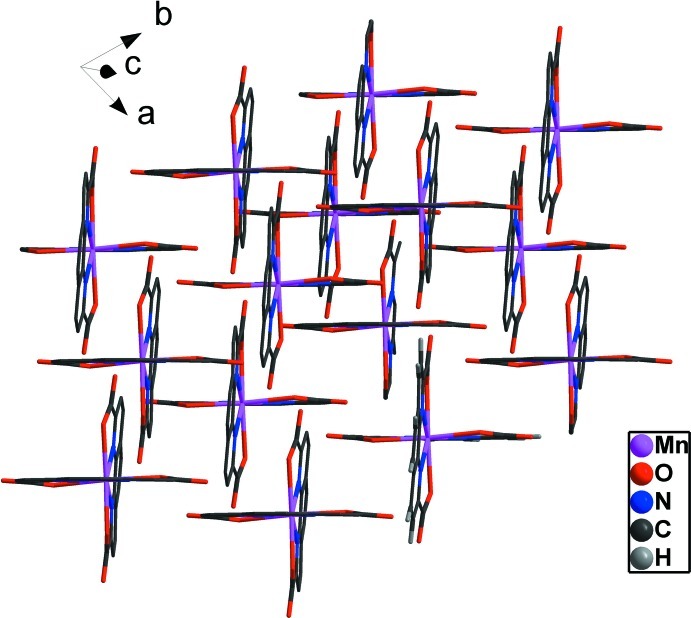
The off-set π-π stacking interaction in the distance of 3.553 (2) Å between phenda2- groups from the neighboring units (the H-atoms on the phenda2- are omitted for clarity)

**Fig. 3.**
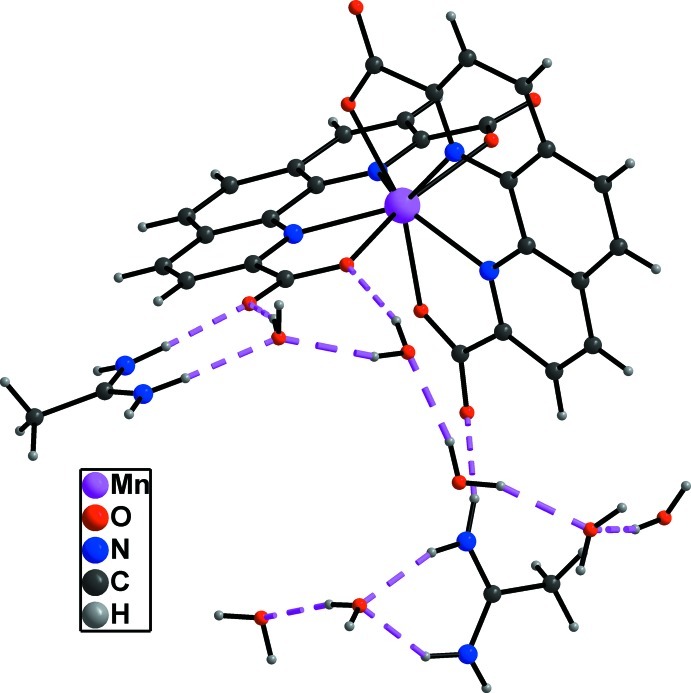
A perspective view of hydrogen bonding interactions shown as dashed lines.

**Fig. 4.**
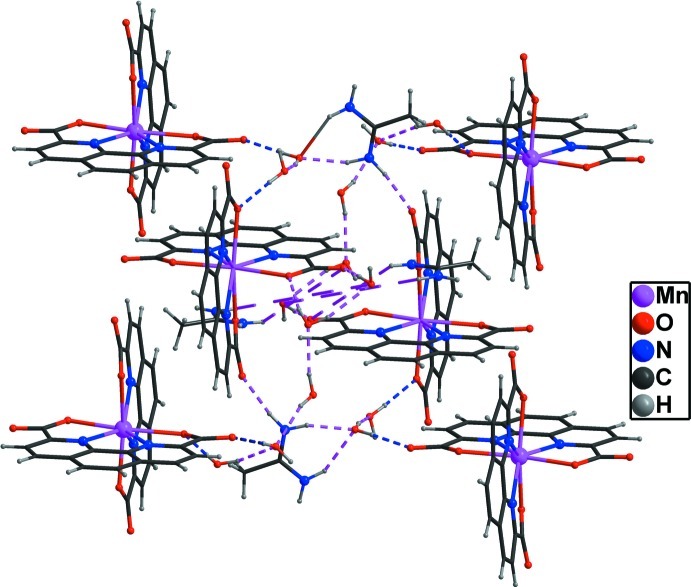
The three-dimensional framework of the title compound through intermolecular π-π stacking interactions and all hydrogen bonds.

## supplementary crystallographic information

### Comment

1,10-Phenanthroline and its substituted derivatives have versatile chelating and bridging capability(Kaes *et al.*, 2000; Albores & Rentschler, 2008) which have played an important role in the development of coordination chemistry while the oxime, similar to the cyanide, can link two magnetic moment carriers with the shortest pairwise exchange pathway (Sreerama & Pal, 2004). Preliminary study on the coordination chemistry of 1,10-phenanthroline-2,9-dicarboxylate (H_2_phenda) found that it chelates the metal ions such as Ca(II), Cu(II), Th(III), Eu(III) and Tb(III) as a tridentate or tetradentate ligand by the phenanthroline and one or both of the oxygen atoms on the carboxylate groups (Dean *et al.*, 2008; Gephart *et al.*, 2008; Moghimi *et al.*, 2005; Fan *et al.*, 2008). We report here the crystal structure of the title compound, (I).

The title compound is ionic and contains discrete 1,10-phenanthroline- 2,9-dicarboxylate manganese(II) anions, ethanamidinium cations and water molecules (Fig. 1). The Mn atom is coordinated by four N atoms and four O atoms from two 1,10-phenanthroline-2,9-dicarboxylate ligands in a distorted dodecahedron geometry.

In the crystal structure, intermolecular π-π interactions (Figure 2) between the neighboring aromatic rings of phenda ligands link the molecules into an infinite layer. The centroid to centroid distance between pyridine rings is 3.553 (2) Å. The crystal packing is further stabilized by N—H···O and O—H···O hydrogen bonds (Table 1 and Figure 3), which link the layers into a three dimensional framework (Figure 4).

### Experimental

Hydrothermal treatment of manganese chloride tetrahydrate (0.2 mmol, 0.039 g), 1,10-phenanthroline-2,9-dicarboxylate (0.2 mmol, 0.054 g) synthesized according to C. J. Chandler *et al.* (1981), triethylamine (0.080 g, 0.4 mmol), and methanol/acetonitrile (15 ml,V/V = 5/1) over 72 h at 418 K yielded light yellow block-shaped crystals.

### Refinement

Diffraction data for compound I were recorded on Oxford Diffraction Gemini *R* CCD diffractometer at 150 (2) K. The data collection routine, unit cell refinement, and data processing were carried out with the program *CrysAlis PRO* for I.

All H atoms were placed in idealized positions, and were refined using as riding model with C—H distances of 0.95, 0.98, 0.90 and 0.85 Å, for aryl, methyl, amido and water, respectively, with *U*_iso_(H) = 1.5*U*_eq_ (methyl C-atoms) and 1.2*U*_eq_(non-methyl C-atoms). The hightest peak is located 1.13 Å from O5W and the deepest hole is located 0.68 Å from N5.

### Crystal data

**Table:**

(C_2_H_7_N_2_)_2_[Mn(C_14_H_6_N_2_O_4_)_2_]·7H_2_O	*Z* = 2
*M**_r_* = 831.66	*F*(000) = 866
Triclinic, *P*1	*D*_x_ = 1.522 Mg m^−^^3^
Hall symbol: -P 1	Cu *K*α radiation, λ = 1.54178 Å
*a* = 9.6330 (6) Å	Cell parameters from 2456 reflections
*b* = 13.8174 (7) Å	θ = 3.8–60.5°
*c* = 15.4828 (8) Å	µ = 3.69 mm^−^^1^
α = 66.151 (5)°	*T* = 150 K
β = 78.949 (5)°	Block, yellow
γ = 75.397 (5)°	0.29 × 0.26 × 0.16 mm
*V* = 1814.56 (19) Å^3^	

### Data collection

**Table:**

Agilent Gemini S Ultra CCD diffractometer	5310 independent reflections
Radiation source: Ultra (Cu) X-ray Source	4336 reflections with *I* > 2σ(*I*)
Mirror monochromator	*R*_int_ = 0.024
ω and ψ scan	θ_max_ = 60.0°, θ_min_ = 3.1°
Absorption correction: multi-scan (Blessing, 1995)	*h* = −10→10
*T*_min_ = 0.395, *T*_max_ = 0.554	*k* = −15→15
10581 measured reflections	*l* = −17→17

### Refinement

**Table:**

Refinement on *F*^2^	Primary atom site location: structure-invariant direct methods
Least-squares matrix: full	Secondary atom site location: difference Fourier map
*R*[*F*^2^ > 2σ(*F*^2^)] = 0.037	Hydrogen site location: inferred from neighbouring sites
*wR*(*F*^2^) = 0.102	H-atom parameters constrained
*S* = 0.95	*w* = 1/[σ^2^(*F*_o_^2^) + (0.0694*P*)^2^] where *P* = (*F*_o_^2^ + 2*F*_c_^2^)/3
5310 reflections	(Δ/σ)_max_ < 0.001
507 parameters	Δρ_max_ = 0.49 e Å^−^^3^
0 restraints	Δρ_min_ = −0.38 e Å^−^^3^

### Special details

**Table:**

Geometry. All e.s.d.'s (except the e.s.d. in the dihedral angle between two l.s. planes) are estimated using the full covariance matrix. The cell e.s.d.'s are taken into account individually in the estimation of e.s.d.'s in distances, angles and torsion angles; correlations between e.s.d.'s in cell parameters are only used when they are defined by crystal symmetry. An approximate (isotropic) treatment of cell e.s.d.'s is used for estimating e.s.d.'s involving l.s. planes.
Refinement. Refinement of *F*^2^ against ALL reflections. The weighted *R*-factor *wR* and goodness of fit *S* are based on *F*^2^, conventional *R*-factors *R* are based on *F*, with *F* set to zero for negative *F*^2^. The threshold expression of *F*^2^ > σ(*F*^2^) is used only for calculating *R*-factors(gt) *etc*. and is not relevant to the choice of reflections for refinement. *R*-factors based on *F*^2^ are statistically about twice as large as those based on *F*, and *R*- factors based on ALL data will be even larger.

### Fractional atomic coordinates and isotropic or equivalent isotropic displacement parameters (Å^2^)

**Table:**

	*x*	*y*	*z*	*U*_iso_*/*U*_eq_	
Mn1	0.38224 (4)	−0.32469 (3)	−0.30135 (3)	0.02456 (13)	
O1	0.62034 (19)	−0.44480 (14)	−0.29790 (12)	0.0315 (4)	
O2	0.7512 (2)	−0.60690 (15)	−0.22274 (14)	0.0405 (5)	
O3	0.19195 (19)	−0.18187 (14)	−0.36856 (13)	0.0322 (4)	
O4	−0.0144 (2)	−0.07603 (17)	−0.34049 (16)	0.0526 (6)	
O5	0.4381 (2)	−0.21686 (15)	−0.22559 (12)	0.0342 (4)	
O6	0.5792 (2)	−0.10790 (16)	−0.23117 (14)	0.0429 (5)	
O7	0.27142 (19)	−0.45336 (14)	−0.31506 (12)	0.0316 (4)	
O8	0.2046 (2)	−0.51264 (15)	−0.41261 (13)	0.0352 (4)	
N1	0.4178 (2)	−0.46684 (16)	−0.15591 (14)	0.0247 (5)	
N2	0.1863 (2)	−0.31304 (16)	−0.19121 (15)	0.0266 (5)	
N3	0.5253 (2)	−0.20108 (16)	−0.39983 (14)	0.0249 (5)	
N4	0.4184 (2)	−0.32404 (16)	−0.45354 (14)	0.0238 (5)	
C1	0.5334 (3)	−0.5458 (2)	−0.14235 (18)	0.0275 (6)	
C2	0.5504 (3)	−0.6328 (2)	−0.05494 (19)	0.0327 (6)	
H2	0.6323	−0.6896	−0.0479	0.039*	
C3	0.4479 (3)	−0.6348 (2)	0.01978 (19)	0.0361 (7)	
H3	0.4594	−0.6924	0.0796	0.043*	
C4	0.3259 (3)	−0.5520 (2)	0.00840 (19)	0.0328 (6)	
C5	0.3154 (3)	−0.4703 (2)	−0.08246 (17)	0.0264 (6)	
C6	0.1906 (3)	−0.3847 (2)	−0.10117 (18)	0.0276 (6)	
C7	0.0811 (3)	−0.3796 (2)	−0.0278 (2)	0.0355 (7)	
C8	−0.0404 (3)	−0.2954 (2)	−0.0534 (2)	0.0398 (7)	
H8	−0.1181	−0.2882	−0.0069	0.048*	
C9	−0.0455 (3)	−0.2239 (2)	−0.1460 (2)	0.0392 (7)	
H9	−0.1277	−0.1681	−0.1643	0.047*	
C10	0.0723 (3)	−0.2344 (2)	−0.2135 (2)	0.0317 (6)	
C11	0.2133 (3)	−0.5429 (2)	0.08190 (19)	0.0386 (7)	
H11	0.2200	−0.5962	0.1442	0.046*	
C12	0.0976 (3)	−0.4606 (2)	0.0651 (2)	0.0398 (7)	
H12	0.0263	−0.4567	0.1159	0.048*	
C13	0.6447 (3)	−0.5317 (2)	−0.22789 (19)	0.0280 (6)	
C14	0.0830 (3)	−0.1565 (2)	−0.3165 (2)	0.0333 (6)	
C15	0.5761 (3)	−0.1406 (2)	−0.37003 (18)	0.0271 (6)	
C16	0.6643 (3)	−0.0680 (2)	−0.4304 (2)	0.0331 (6)	
H16	0.6989	−0.0256	−0.4069	0.040*	
C17	0.7004 (3)	−0.0583 (2)	−0.5229 (2)	0.0337 (6)	
H17	0.7605	−0.0096	−0.5639	0.040*	
C18	0.6479 (3)	−0.1210 (2)	−0.55703 (19)	0.0299 (6)	
C19	0.5597 (3)	−0.19174 (19)	−0.49131 (18)	0.0250 (5)	
C20	0.5006 (3)	−0.2583 (2)	−0.52032 (17)	0.0248 (5)	
C21	0.5305 (3)	−0.2531 (2)	−0.61455 (18)	0.0292 (6)	
C22	0.4655 (3)	−0.3189 (2)	−0.63757 (19)	0.0326 (6)	
H22	0.4809	−0.3179	−0.7004	0.039*	
C23	0.3805 (3)	−0.3840 (2)	−0.57014 (18)	0.0308 (6)	
H23	0.3358	−0.4279	−0.5857	0.037*	
C24	0.3596 (3)	−0.3856 (2)	−0.47696 (18)	0.0264 (6)	
C25	0.6768 (3)	−0.1183 (2)	−0.65205 (19)	0.0333 (6)	
H25	0.7367	−0.0717	−0.6969	0.040*	
C26	0.6210 (3)	−0.1805 (2)	−0.67956 (19)	0.0349 (6)	
H26	0.6420	−0.1761	−0.7434	0.042*	
C27	0.5275 (3)	−0.1559 (2)	−0.26719 (19)	0.0301 (6)	
C28	0.2712 (3)	−0.4568 (2)	−0.39516 (18)	0.0276 (6)	
N5	0.8010 (3)	−0.8300 (2)	−0.1744 (2)	0.0640 (8)	
H5A	0.7521	−0.8547	−0.2026	0.077*	
H5B	0.7762	−0.7619	−0.1765	0.077*	
N6	0.9723 (3)	−0.9812 (2)	−0.1703 (2)	0.0604 (8)	
H6A	0.9292	−0.9846	−0.2154	0.073*	
H6B	1.0600	−1.0209	−0.1545	0.073*	
C29	0.9287 (4)	−0.8931 (3)	−0.1516 (2)	0.0517 (8)	
C30	1.0219 (4)	−0.8639 (3)	−0.1027 (3)	0.0615 (10)	
H30A	0.9621	−0.8381	−0.0545	0.092*	
H30B	1.0707	−0.8069	−0.1493	0.092*	
H30C	1.0939	−0.9275	−0.0721	0.092*	
N7	0.1675 (2)	−0.59460 (18)	−0.54617 (16)	0.0329 (5)	
H7A	0.1679	−0.5739	−0.4981	0.039*	
H7B	0.2252	−0.5741	−0.6008	0.039*	
N8	0.0118 (2)	−0.70915 (18)	−0.45609 (16)	0.0349 (5)	
H8A	0.0121	−0.6954	−0.4041	0.042*	
H8B	−0.0380	−0.7590	−0.4501	0.042*	
C31	0.0874 (3)	−0.6628 (2)	−0.53660 (19)	0.0309 (6)	
C32	0.0816 (3)	−0.6870 (2)	−0.6215 (2)	0.0375 (7)	
H32A	0.1707	−0.6759	−0.6639	0.056*	
H32B	−0.0011	−0.6389	−0.6551	0.056*	
H32C	0.0715	−0.7621	−0.6010	0.056*	
O1W	0.7167 (2)	−0.93913 (18)	−0.26699 (16)	0.0517 (6)	
H1WB	0.6725	−0.9894	−0.2567	0.062*	
H1WA	0.7559	−0.9119	−0.3232	0.062*	
O2W	0.8697 (2)	−0.87175 (15)	−0.44941 (13)	0.0374 (5)	
H2WA	0.9239	−0.9339	−0.4315	0.045*	
H2WB	0.8510	−0.8574	−0.5052	0.045*	
O3W	0.0311 (2)	−0.64852 (16)	−0.29789 (14)	0.0433 (5)	
H3WA	0.0748	−0.5962	−0.3280	0.052*	
H3WB	−0.0544	−0.6172	−0.2865	0.052*	
O4W	0.2349 (2)	−0.65284 (17)	−0.17187 (14)	0.0455 (5)	
H4WA	0.1802	−0.6675	−0.2001	0.055*	
H4WB	0.2411	−0.5878	−0.2062	0.055*	
O5W	0.4446 (4)	−0.8419 (2)	−0.1000 (2)	0.0860 (10)	
H5WA	0.3813	−0.7841	−0.1141	0.103*	
H5WB	0.4500	−0.8624	−0.0409	0.103*	
O6W	0.5485 (3)	−0.94350 (19)	0.07739 (16)	0.0579 (6)	
H6WA	0.5041	−0.9299	0.1255	0.069*	
H6WB	0.5758	−1.0060	0.0752	0.069*	
O7W	0.7721 (2)	−0.87078 (19)	0.10478 (17)	0.0560 (6)	
H7WA	0.7217	−0.8988	0.0853	0.067*	
H7WB	0.7133	−0.8399	0.1386	0.067*	

### Atomic displacement parameters (Å^2^)

**Table:**

	*U*^11^	*U*^22^	*U*^33^	*U*^12^	*U*^13^	*U*^23^
Mn1	0.0283 (2)	0.0252 (2)	0.0204 (2)	−0.00653 (17)	−0.00202 (16)	−0.00818 (17)
O1	0.0312 (10)	0.0301 (10)	0.0291 (10)	−0.0049 (8)	−0.0017 (8)	−0.0083 (9)
O2	0.0352 (11)	0.0331 (11)	0.0472 (12)	0.0015 (9)	−0.0024 (9)	−0.0146 (9)
O3	0.0336 (10)	0.0295 (10)	0.0315 (10)	−0.0048 (8)	−0.0076 (8)	−0.0084 (8)
O4	0.0449 (13)	0.0370 (12)	0.0569 (14)	0.0085 (10)	−0.0079 (10)	−0.0069 (10)
O5	0.0441 (11)	0.0353 (11)	0.0285 (10)	−0.0152 (9)	−0.0045 (8)	−0.0127 (9)
O6	0.0539 (13)	0.0485 (12)	0.0396 (11)	−0.0203 (10)	−0.0067 (9)	−0.0230 (10)
O7	0.0396 (11)	0.0334 (10)	0.0273 (10)	−0.0131 (8)	−0.0031 (8)	−0.0134 (8)
O8	0.0372 (11)	0.0407 (11)	0.0379 (11)	−0.0153 (9)	−0.0038 (8)	−0.0204 (9)
N1	0.0286 (11)	0.0243 (11)	0.0247 (11)	−0.0051 (9)	−0.0060 (9)	−0.0113 (9)
N2	0.0281 (12)	0.0257 (11)	0.0289 (12)	−0.0063 (9)	−0.0039 (9)	−0.0122 (10)
N3	0.0272 (11)	0.0215 (11)	0.0269 (11)	−0.0028 (9)	−0.0064 (9)	−0.0095 (9)
N4	0.0249 (11)	0.0221 (11)	0.0260 (11)	−0.0026 (9)	−0.0060 (9)	−0.0102 (9)
C1	0.0333 (14)	0.0247 (14)	0.0293 (14)	−0.0079 (11)	−0.0086 (11)	−0.0112 (12)
C2	0.0386 (16)	0.0259 (14)	0.0316 (15)	−0.0056 (12)	−0.0113 (12)	−0.0057 (12)
C3	0.0480 (18)	0.0325 (15)	0.0273 (15)	−0.0142 (13)	−0.0128 (13)	−0.0031 (12)
C4	0.0425 (16)	0.0357 (16)	0.0262 (14)	−0.0176 (13)	−0.0055 (12)	−0.0108 (12)
C5	0.0333 (14)	0.0277 (14)	0.0236 (13)	−0.0123 (11)	−0.0040 (11)	−0.0108 (11)
C6	0.0319 (14)	0.0295 (14)	0.0275 (14)	−0.0116 (11)	−0.0003 (11)	−0.0149 (12)
C7	0.0378 (16)	0.0438 (17)	0.0356 (16)	−0.0180 (13)	0.0044 (12)	−0.0233 (14)
C8	0.0350 (16)	0.0457 (18)	0.0457 (18)	−0.0128 (14)	0.0090 (13)	−0.0269 (15)
C9	0.0321 (16)	0.0356 (16)	0.0527 (19)	−0.0052 (12)	0.0012 (13)	−0.0225 (15)
C10	0.0291 (15)	0.0290 (15)	0.0419 (16)	−0.0047 (12)	−0.0056 (12)	−0.0181 (13)
C11	0.0515 (19)	0.0471 (18)	0.0229 (14)	−0.0254 (15)	−0.0001 (12)	−0.0110 (13)
C12	0.0467 (18)	0.0498 (19)	0.0296 (15)	−0.0222 (15)	0.0081 (13)	−0.0192 (14)
C13	0.0270 (14)	0.0278 (15)	0.0337 (15)	−0.0054 (12)	−0.0062 (11)	−0.0145 (13)
C14	0.0336 (16)	0.0270 (15)	0.0402 (16)	−0.0053 (12)	−0.0077 (13)	−0.0122 (13)
C15	0.0262 (13)	0.0239 (13)	0.0318 (14)	−0.0022 (11)	−0.0074 (11)	−0.0105 (11)
C16	0.0336 (15)	0.0295 (15)	0.0410 (17)	−0.0105 (12)	−0.0068 (12)	−0.0142 (13)
C17	0.0310 (15)	0.0300 (15)	0.0383 (16)	−0.0107 (12)	−0.0022 (12)	−0.0086 (13)
C18	0.0261 (14)	0.0261 (14)	0.0339 (15)	−0.0027 (11)	−0.0038 (11)	−0.0086 (12)
C19	0.0235 (13)	0.0212 (13)	0.0280 (14)	−0.0010 (10)	−0.0044 (10)	−0.0079 (11)
C20	0.0236 (13)	0.0240 (13)	0.0248 (13)	−0.0001 (10)	−0.0065 (10)	−0.0080 (11)
C21	0.0291 (14)	0.0303 (15)	0.0259 (14)	0.0001 (11)	−0.0052 (11)	−0.0106 (12)
C22	0.0360 (15)	0.0380 (16)	0.0259 (14)	−0.0030 (12)	−0.0048 (12)	−0.0160 (13)
C23	0.0354 (15)	0.0321 (15)	0.0299 (14)	−0.0059 (12)	−0.0057 (12)	−0.0160 (13)
C24	0.0267 (13)	0.0246 (13)	0.0293 (14)	−0.0004 (11)	−0.0073 (11)	−0.0122 (11)
C25	0.0307 (15)	0.0349 (15)	0.0281 (14)	−0.0090 (12)	0.0030 (11)	−0.0066 (12)
C26	0.0356 (15)	0.0404 (17)	0.0251 (14)	−0.0062 (13)	0.0006 (12)	−0.0111 (13)
C27	0.0347 (15)	0.0261 (14)	0.0327 (14)	−0.0051 (12)	−0.0106 (12)	−0.0115 (12)
C28	0.0268 (14)	0.0242 (14)	0.0319 (15)	−0.0005 (11)	−0.0062 (11)	−0.0118 (12)
N5	0.070 (2)	0.0449 (17)	0.083 (2)	0.0005 (15)	−0.0239 (17)	−0.0293 (16)
N6	0.0657 (19)	0.0425 (16)	0.081 (2)	0.0048 (14)	−0.0284 (16)	−0.0308 (16)
C29	0.055 (2)	0.046 (2)	0.054 (2)	−0.0131 (16)	−0.0062 (16)	−0.0152 (16)
C30	0.073 (3)	0.059 (2)	0.059 (2)	−0.0178 (19)	−0.0177 (19)	−0.0208 (19)
N7	0.0352 (13)	0.0374 (13)	0.0304 (12)	−0.0120 (11)	−0.0006 (10)	−0.0155 (11)
N8	0.0334 (13)	0.0360 (13)	0.0359 (13)	−0.0091 (10)	−0.0043 (10)	−0.0123 (11)
C31	0.0266 (14)	0.0299 (15)	0.0345 (15)	−0.0010 (12)	−0.0046 (12)	−0.0124 (12)
C32	0.0351 (16)	0.0399 (16)	0.0428 (17)	−0.0070 (13)	−0.0063 (13)	−0.0200 (14)
O1W	0.0516 (13)	0.0528 (14)	0.0579 (14)	−0.0179 (11)	0.0000 (11)	−0.0261 (12)
O2W	0.0433 (11)	0.0324 (10)	0.0354 (10)	−0.0074 (9)	−0.0120 (9)	−0.0081 (9)
O3W	0.0378 (11)	0.0422 (12)	0.0452 (12)	−0.0079 (9)	0.0024 (9)	−0.0145 (10)
O4W	0.0535 (13)	0.0419 (12)	0.0386 (11)	−0.0154 (10)	−0.0028 (10)	−0.0096 (10)
O5W	0.135 (3)	0.0618 (17)	0.0708 (18)	0.0146 (17)	−0.0524 (18)	−0.0365 (15)
O6W	0.0748 (16)	0.0636 (15)	0.0467 (13)	−0.0144 (12)	−0.0092 (11)	−0.0304 (12)
O7W	0.0481 (13)	0.0637 (15)	0.0654 (15)	−0.0037 (11)	−0.0068 (11)	−0.0373 (13)

### Geometric parameters (Å, º)

**Table:**

Mn1—N2	2.309 (2)	C17—H17	0.9500
Mn1—N4	2.312 (2)	C18—C19	1.412 (4)
Mn1—N3	2.327 (2)	C18—C25	1.431 (4)
Mn1—N1	2.329 (2)	C19—C20	1.439 (4)
Mn1—O3	2.3630 (18)	C20—C21	1.407 (4)
Mn1—O7	2.3863 (18)	C21—C22	1.407 (4)
Mn1—O5	2.4382 (17)	C21—C26	1.438 (4)
Mn1—O1	2.4645 (18)	C22—C23	1.361 (4)
O1—C13	1.255 (3)	C22—H22	0.9500
O2—C13	1.251 (3)	C23—C24	1.410 (4)
O3—C14	1.262 (3)	C23—H23	0.9500
O4—C14	1.237 (3)	C24—C28	1.517 (4)
O5—C27	1.258 (3)	C25—C26	1.351 (4)
O6—C27	1.253 (3)	C25—H25	0.9500
O7—C28	1.261 (3)	C26—H26	0.9500
O8—C28	1.250 (3)	N5—C29	1.332 (4)
N1—C1	1.329 (3)	N5—H5A	0.9000
N1—C5	1.348 (3)	N5—H5B	0.9000
N2—C10	1.321 (3)	N6—C29	1.313 (4)
N2—C6	1.345 (3)	N6—H6A	0.9000
N3—C15	1.325 (3)	N6—H6B	0.9000
N3—C19	1.351 (3)	C29—C30	1.493 (5)
N4—C24	1.322 (3)	C30—H30A	0.9800
N4—C20	1.345 (3)	C30—H30B	0.9800
C1—C2	1.405 (4)	C30—H30C	0.9800
C1—C13	1.514 (4)	N7—C31	1.310 (4)
C2—C3	1.365 (4)	N7—H7A	0.8999
C2—H2	0.9500	N7—H7B	0.9000
C3—C4	1.400 (4)	N8—C31	1.317 (4)
C3—H3	0.9500	N8—H8A	0.9000
C4—C5	1.406 (4)	N8—H8B	0.8999
C4—C11	1.436 (4)	C31—C32	1.495 (4)
C5—C6	1.439 (4)	C32—H32A	0.9800
C6—C7	1.405 (4)	C32—H32B	0.9800
C7—C8	1.414 (4)	C32—H32C	0.9800
C7—C12	1.430 (4)	O1W—H1WB	0.8501
C8—C9	1.375 (4)	O1W—H1WA	0.8499
C8—H8	0.9500	O2W—H2WA	0.8500
C9—C10	1.409 (4)	O2W—H2WB	0.8499
C9—H9	0.9500	O3W—H3WA	0.8498
C10—C14	1.523 (4)	O3W—H3WB	0.8501
C11—C12	1.354 (4)	O4W—H4WA	0.8499
C11—H11	0.9500	O4W—H4WB	0.8500
C12—H12	0.9500	O5W—H5WA	0.8500
C15—C16	1.403 (4)	O5W—H5WB	0.8499
C15—C27	1.514 (4)	O6W—H6WA	0.8501
C16—C17	1.368 (4)	O6W—H6WB	0.8499
C16—H16	0.9500	O7W—H7WA	0.8500
C17—C18	1.407 (4)	O7W—H7WB	0.8501
			
N2—Mn1—N4	136.11 (7)	O4—C14—O3	126.9 (3)
N2—Mn1—N3	132.09 (7)	O4—C14—C10	117.4 (2)
N4—Mn1—N3	69.49 (7)	O3—C14—C10	115.6 (2)
N2—Mn1—N1	69.13 (7)	N3—C15—C16	122.0 (2)
N4—Mn1—N1	130.55 (7)	N3—C15—C27	113.8 (2)
N3—Mn1—N1	131.49 (7)	C16—C15—C27	124.1 (2)
N2—Mn1—O3	67.82 (7)	C17—C16—C15	119.9 (2)
N4—Mn1—O3	80.79 (7)	C17—C16—H16	120.0
N3—Mn1—O3	83.18 (7)	C15—C16—H16	120.0
N1—Mn1—O3	136.69 (7)	C16—C17—C18	119.6 (3)
N2—Mn1—O7	83.15 (7)	C16—C17—H17	120.2
N4—Mn1—O7	67.47 (7)	C18—C17—H17	120.2
N3—Mn1—O7	136.91 (7)	C17—C18—C19	116.6 (2)
N1—Mn1—O7	78.60 (7)	C17—C18—C25	125.2 (3)
O3—Mn1—O7	91.77 (6)	C19—C18—C25	118.2 (2)
N2—Mn1—O5	75.67 (7)	N3—C19—C18	123.4 (2)
N4—Mn1—O5	135.37 (7)	N3—C19—C20	116.3 (2)
N3—Mn1—O5	66.19 (7)	C18—C19—C20	120.3 (2)
N1—Mn1—O5	85.00 (7)	N4—C20—C21	123.3 (2)
O3—Mn1—O5	88.98 (6)	N4—C20—C19	116.7 (2)
O7—Mn1—O5	156.79 (6)	C21—C20—C19	120.0 (2)
N2—Mn1—O1	135.12 (7)	C22—C21—C20	116.3 (3)
N4—Mn1—O1	79.20 (6)	C22—C21—C26	125.4 (2)
N3—Mn1—O1	79.36 (7)	C20—C21—C26	118.3 (2)
N1—Mn1—O1	66.09 (6)	C23—C22—C21	120.2 (2)
O3—Mn1—O1	157.04 (6)	C23—C22—H22	119.9
O7—Mn1—O1	90.89 (6)	C21—C22—H22	119.9
O5—Mn1—O1	97.40 (6)	C22—C23—C24	119.4 (2)
C13—O1—Mn1	119.48 (16)	C22—C23—H23	120.3
C14—O3—Mn1	120.52 (16)	C24—C23—H23	120.3
C27—O5—Mn1	120.15 (16)	N4—C24—C23	121.7 (3)
C28—O7—Mn1	120.22 (17)	N4—C24—C28	114.0 (2)
C1—N1—C5	118.4 (2)	C23—C24—C28	124.3 (2)
C1—N1—Mn1	122.99 (17)	C26—C25—C18	121.6 (3)
C5—N1—Mn1	118.63 (16)	C26—C25—H25	119.2
C10—N2—C6	119.0 (2)	C18—C25—H25	119.2
C10—N2—Mn1	121.43 (18)	C25—C26—C21	121.6 (2)
C6—N2—Mn1	119.54 (16)	C25—C26—H26	119.2
C15—N3—C19	118.5 (2)	C21—C26—H26	119.2
C15—N3—Mn1	123.05 (17)	O6—C27—O5	126.0 (3)
C19—N3—Mn1	118.41 (16)	O6—C27—C15	118.0 (3)
C24—N4—C20	119.1 (2)	O5—C27—C15	116.0 (2)
C24—N4—Mn1	121.88 (17)	O8—C28—O7	126.0 (3)
C20—N4—Mn1	119.00 (15)	O8—C28—C24	118.0 (2)
N1—C1—C2	122.1 (2)	O7—C28—C24	116.0 (2)
N1—C1—C13	114.2 (2)	C29—N5—H5A	113.1
C2—C1—C13	123.7 (2)	C29—N5—H5B	123.4
C3—C2—C1	119.3 (3)	H5A—N5—H5B	121.6
C3—C2—H2	120.4	C29—N6—H6A	116.3
C1—C2—H2	120.4	C29—N6—H6B	117.8
C2—C3—C4	120.1 (3)	H6A—N6—H6B	122.1
C2—C3—H3	120.0	N6—C29—N5	120.7 (3)
C4—C3—H3	120.0	N6—C29—C30	119.8 (3)
C3—C4—C5	116.7 (3)	N5—C29—C30	119.5 (3)
C3—C4—C11	125.5 (3)	C29—C30—H30A	109.5
C5—C4—C11	117.9 (3)	C29—C30—H30B	109.5
N1—C5—C4	123.4 (2)	H30A—C30—H30B	109.5
N1—C5—C6	116.4 (2)	C29—C30—H30C	109.5
C4—C5—C6	120.2 (2)	H30A—C30—H30C	109.5
N2—C6—C7	123.6 (2)	H30B—C30—H30C	109.5
N2—C6—C5	116.1 (2)	C31—N7—H7A	120.3
C7—C6—C5	120.2 (2)	C31—N7—H7B	116.6
C6—C7—C8	116.4 (3)	H7A—N7—H7B	122.9
C6—C7—C12	118.5 (3)	C31—N8—H8A	121.6
C8—C7—C12	125.0 (3)	C31—N8—H8B	121.0
C9—C8—C7	119.6 (3)	H8A—N8—H8B	117.3
C9—C8—H8	120.2	N7—C31—N8	122.6 (2)
C7—C8—H8	120.2	N7—C31—C32	117.9 (2)
C8—C9—C10	119.3 (3)	N8—C31—C32	119.6 (2)
C8—C9—H9	120.4	C31—C32—H32A	109.5
C10—C9—H9	120.4	C31—C32—H32B	109.5
N2—C10—C9	122.0 (3)	H32A—C32—H32B	109.5
N2—C10—C14	113.9 (2)	C31—C32—H32C	109.5
C9—C10—C14	124.1 (2)	H32A—C32—H32C	109.5
C12—C11—C4	122.0 (3)	H32B—C32—H32C	109.5
C12—C11—H11	119.0	H1WB—O1W—H1WA	115.9
C4—C11—H11	119.0	H2WA—O2W—H2WB	107.1
C11—C12—C7	121.1 (3)	H3WA—O3W—H3WB	103.0
C11—C12—H12	119.4	H4WA—O4W—H4WB	103.6
C7—C12—H12	119.4	H5WA—O5W—H5WB	105.4
O2—C13—O1	126.1 (2)	H6WA—O6W—H6WB	125.3
O2—C13—C1	117.7 (2)	H7WA—O7W—H7WB	105.6
O1—C13—C1	116.2 (2)		
			
N2—Mn1—O1—C13	13.5 (2)	C3—C4—C5—N1	−2.4 (4)
N4—Mn1—O1—C13	−134.56 (18)	C11—C4—C5—N1	176.4 (2)
N3—Mn1—O1—C13	154.53 (18)	C3—C4—C5—C6	177.5 (2)
N1—Mn1—O1—C13	9.39 (17)	C11—C4—C5—C6	−3.7 (4)
O3—Mn1—O1—C13	−164.36 (18)	C10—N2—C6—C7	−1.4 (4)
O7—Mn1—O1—C13	−67.71 (18)	Mn1—N2—C6—C7	175.47 (19)
O5—Mn1—O1—C13	90.55 (18)	C10—N2—C6—C5	177.9 (2)
N2—Mn1—O3—C14	7.74 (18)	Mn1—N2—C6—C5	−5.2 (3)
N4—Mn1—O3—C14	156.4 (2)	N1—C5—C6—N2	3.0 (3)
N3—Mn1—O3—C14	−133.32 (19)	C4—C5—C6—N2	−176.8 (2)
N1—Mn1—O3—C14	14.4 (2)	N1—C5—C6—C7	−177.6 (2)
O7—Mn1—O3—C14	89.61 (19)	C4—C5—C6—C7	2.6 (4)
O5—Mn1—O3—C14	−67.18 (19)	N2—C6—C7—C8	1.9 (4)
O1—Mn1—O3—C14	−173.92 (18)	C5—C6—C7—C8	−177.4 (2)
N2—Mn1—O5—C27	−158.4 (2)	N2—C6—C7—C12	179.9 (2)
N4—Mn1—O5—C27	−15.3 (2)	C5—C6—C7—C12	0.5 (4)
N3—Mn1—O5—C27	−8.08 (18)	C6—C7—C8—C9	−0.3 (4)
N1—Mn1—O5—C27	131.87 (19)	C12—C7—C8—C9	−178.1 (3)
O3—Mn1—O5—C27	−91.07 (19)	C7—C8—C9—C10	−1.6 (4)
O7—Mn1—O5—C27	176.82 (18)	C6—N2—C10—C9	−0.7 (4)
O1—Mn1—O5—C27	66.80 (19)	Mn1—N2—C10—C9	−177.49 (19)
N2—Mn1—O7—C28	140.83 (18)	C6—N2—C10—C14	177.9 (2)
N4—Mn1—O7—C28	−5.86 (17)	Mn1—N2—C10—C14	1.1 (3)
N3—Mn1—O7—C28	−8.5 (2)	C8—C9—C10—N2	2.2 (4)
N1—Mn1—O7—C28	−149.16 (18)	C8—C9—C10—C14	−176.3 (3)
O3—Mn1—O7—C28	73.41 (18)	C3—C4—C11—C12	−179.4 (3)
O5—Mn1—O7—C28	164.95 (17)	C5—C4—C11—C12	1.9 (4)
O1—Mn1—O7—C28	−83.78 (18)	C4—C11—C12—C7	1.2 (4)
N2—Mn1—N1—C1	176.7 (2)	C6—C7—C12—C11	−2.4 (4)
N4—Mn1—N1—C1	43.1 (2)	C8—C7—C12—C11	175.3 (3)
N3—Mn1—N1—C1	−55.0 (2)	Mn1—O1—C13—O2	168.8 (2)
O3—Mn1—N1—C1	170.03 (16)	Mn1—O1—C13—C1	−11.0 (3)
O7—Mn1—N1—C1	89.71 (19)	N1—C1—C13—O2	−174.7 (2)
O5—Mn1—N1—C1	−106.80 (19)	C2—C1—C13—O2	6.8 (4)
O1—Mn1—N1—C1	−6.42 (17)	N1—C1—C13—O1	5.1 (3)
N2—Mn1—N1—C5	−2.20 (16)	C2—C1—C13—O1	−173.4 (2)
N4—Mn1—N1—C5	−135.73 (16)	Mn1—O3—C14—O4	170.2 (2)
N3—Mn1—N1—C5	126.16 (17)	Mn1—O3—C14—C10	−9.9 (3)
O3—Mn1—N1—C5	−8.8 (2)	N2—C10—C14—O4	−174.3 (2)
O7—Mn1—N1—C5	−89.14 (17)	C9—C10—C14—O4	4.2 (4)
O5—Mn1—N1—C5	74.35 (17)	N2—C10—C14—O3	5.8 (3)
O1—Mn1—N1—C5	174.73 (19)	C9—C10—C14—O3	−175.7 (2)
N4—Mn1—N2—C10	−51.9 (2)	C19—N3—C15—C16	−0.2 (4)
N3—Mn1—N2—C10	53.1 (2)	Mn1—N3—C15—C16	178.83 (18)
N1—Mn1—N2—C10	−179.3 (2)	C19—N3—C15—C27	178.6 (2)
O3—Mn1—N2—C10	−4.16 (18)	Mn1—N3—C15—C27	−2.4 (3)
O7—Mn1—N2—C10	−98.89 (19)	N3—C15—C16—C17	−0.1 (4)
O5—Mn1—N2—C10	90.68 (19)	C27—C15—C16—C17	−178.8 (2)
O1—Mn1—N2—C10	176.76 (17)	C15—C16—C17—C18	0.4 (4)
N4—Mn1—N2—C6	131.34 (17)	C16—C17—C18—C19	−0.3 (4)
N3—Mn1—N2—C6	−123.72 (17)	C16—C17—C18—C25	179.7 (3)
N1—Mn1—N2—C6	3.94 (17)	C15—N3—C19—C18	0.3 (4)
O3—Mn1—N2—C6	179.04 (19)	Mn1—N3—C19—C18	−178.78 (18)
O7—Mn1—N2—C6	84.31 (18)	C15—N3—C19—C20	−179.4 (2)
O5—Mn1—N2—C6	−86.12 (18)	Mn1—N3—C19—C20	1.6 (3)
O1—Mn1—N2—C6	0.0 (2)	C17—C18—C19—N3	−0.1 (4)
N2—Mn1—N3—C15	45.3 (2)	C25—C18—C19—N3	180.0 (2)
N4—Mn1—N3—C15	179.7 (2)	C17—C18—C19—C20	179.6 (2)
N1—Mn1—N3—C15	−53.8 (2)	C25—C18—C19—C20	−0.4 (4)
O3—Mn1—N3—C15	96.98 (19)	C24—N4—C20—C21	−1.5 (4)
O7—Mn1—N3—C15	−177.74 (16)	Mn1—N4—C20—C21	179.14 (18)
O5—Mn1—N3—C15	5.08 (18)	C24—N4—C20—C19	178.9 (2)
O1—Mn1—N3—C15	−97.99 (19)	Mn1—N4—C20—C19	−0.5 (3)
N2—Mn1—N3—C19	−135.64 (16)	N3—C19—C20—N4	−0.7 (3)
N4—Mn1—N3—C19	−1.30 (16)	C18—C19—C20—N4	179.6 (2)
N1—Mn1—N3—C19	125.27 (17)	N3—C19—C20—C21	179.7 (2)
O3—Mn1—N3—C19	−83.99 (17)	C18—C19—C20—C21	0.0 (4)
O7—Mn1—N3—C19	1.3 (2)	N4—C20—C21—C22	2.0 (4)
O5—Mn1—N3—C19	−175.89 (19)	C19—C20—C21—C22	−178.4 (2)
O1—Mn1—N3—C19	81.04 (17)	N4—C20—C21—C26	−179.4 (2)
N2—Mn1—N4—C24	−48.4 (2)	C19—C20—C21—C26	0.2 (4)
N3—Mn1—N4—C24	−178.4 (2)	C20—C21—C22—C23	−0.9 (4)
N1—Mn1—N4—C24	53.9 (2)	C26—C21—C22—C23	−179.4 (2)
O3—Mn1—N4—C24	−92.32 (19)	C21—C22—C23—C24	−0.6 (4)
O7—Mn1—N4—C24	3.49 (17)	C20—N4—C24—C23	−0.1 (4)
O5—Mn1—N4—C24	−171.37 (16)	Mn1—N4—C24—C23	179.22 (17)
O1—Mn1—N4—C24	99.01 (19)	C20—N4—C24—C28	179.3 (2)
N2—Mn1—N4—C20	130.95 (17)	Mn1—N4—C24—C28	−1.4 (3)
N3—Mn1—N4—C20	0.90 (16)	C22—C23—C24—N4	1.2 (4)
N1—Mn1—N4—C20	−126.74 (17)	C22—C23—C24—C28	−178.2 (2)
O3—Mn1—N4—C20	87.01 (17)	C17—C18—C25—C26	−179.3 (3)
O7—Mn1—N4—C20	−177.19 (19)	C19—C18—C25—C26	0.6 (4)
O5—Mn1—N4—C20	8.0 (2)	C18—C25—C26—C21	−0.5 (4)
O1—Mn1—N4—C20	−81.66 (17)	C22—C21—C26—C25	178.5 (3)
C5—N1—C1—C2	1.1 (4)	C20—C21—C26—C25	0.1 (4)
Mn1—N1—C1—C2	−177.75 (18)	Mn1—O5—C27—O6	−170.0 (2)
C5—N1—C1—C13	−177.5 (2)	Mn1—O5—C27—C15	9.8 (3)
Mn1—N1—C1—C13	3.7 (3)	N3—C15—C27—O6	174.7 (2)
N1—C1—C2—C3	−2.5 (4)	C16—C15—C27—O6	−6.5 (4)
C13—C1—C2—C3	175.9 (2)	N3—C15—C27—O5	−5.0 (3)
C1—C2—C3—C4	1.4 (4)	C16—C15—C27—O5	173.7 (2)
C2—C3—C4—C5	0.9 (4)	Mn1—O7—C28—O8	−172.32 (19)
C2—C3—C4—C11	−177.8 (3)	Mn1—O7—C28—C24	7.3 (3)
C1—N1—C5—C4	1.4 (4)	N4—C24—C28—O8	175.7 (2)
Mn1—N1—C5—C4	−179.72 (18)	C23—C24—C28—O8	−4.9 (4)
C1—N1—C5—C6	−178.4 (2)	N4—C24—C28—O7	−3.9 (3)
Mn1—N1—C5—C6	0.5 (3)	C23—C24—C28—O7	175.5 (2)

### Hydrogen-bond geometry (Å, º)

**Table:**

*D*—H···*A*	*D*—H	H···*A*	*D*···*A*	*D*—H···*A*
N5—H5*A*···O1*W*	0.90	1.93	2.792 (4)	159
N5—H5*B*···O2	0.90	1.93	2.797 (3)	161
N6—H6*A*···O1*W*	0.90	2.19	2.938 (4)	140
N6—H6*B*···O7*W*^i^	0.90	1.94	2.830 (3)	169
N7—H7*A*···O8	0.90	1.96	2.840 (3)	167
N7—H7*B*···O1^ii^	0.90	1.93	2.812 (3)	168
N8—H8*A*···O3*W*	0.90	2.05	2.941 (3)	172
N8—H8*B*···O2*W*^iii^	0.90	1.98	2.871 (3)	173
O1*W*—H1*WB*···O6^iv^	0.85	1.93	2.780 (3)	178
O1*W*—H1*WA*···O2*W*	0.85	1.98	2.826 (3)	171
O2*W*—H2*WA*···O4^v^	0.85	1.92	2.709 (3)	154
O2*W*—H2*WB*···O3^ii^	0.85	1.90	2.753 (3)	177
O3*W*—H3*WA*···O8	0.85	1.87	2.687 (3)	161
O3*W*—H3*WB*···O2^iii^	0.85	1.94	2.741 (3)	156
O4*W*—H4*WA*···O3*W*	0.85	2.18	2.996 (3)	161
O4*W*—H4*WB*···O7	0.85	1.98	2.808 (3)	164
O5*W*—H5*WA*···O4*W*	0.85	1.99	2.828 (3)	168
O5*W*—H5*WB*···O6*W*	0.85	1.99	2.783 (3)	154
O6*W*—H6*WA*···O6^vi^	0.85	1.89	2.737 (3)	174
O6*W*—H6*WB*···O5*W*^vii^	0.85	2.03	2.826 (4)	155
O7*W*—H7*WA*···O6*W*	0.85	1.96	2.760 (3)	157
O7*W*—H7*WB*···O5^vi^	0.85	2.02	2.865 (3)	173

Symmetry codes: (i) −*x*+2, −*y*−2, −*z*; (ii) −*x*+1, −*y*−1, −*z*−1; (iii) *x*−1, *y*, *z*; (iv) *x*, *y*−1, *z*; (v) *x*+1, *y*−1, *z*; (vi) −*x*+1, −*y*−1, −*z*; (vii) −*x*+1, −*y*−2, −*z*.
